# Peptide Receptor Radionuclide Therapy (PRRT) Using Actinium-225- and Ac-225/Lutetium-177-Labeled (TANDEM) Somatostatin Receptor Antagonist DOTA-LM3 in Patients with Neuroendocrine Neoplasm: A Retrospective Study Concerning Safety and Survival

**DOI:** 10.3390/cancers17183070

**Published:** 2025-09-19

**Authors:** Elisabetta Perrone, Maria Lucia Calcagni, Lucia Leccisotti, Roberto Moretti, Kriti Ghai, Aleksandr Eismant, Tanay Parkar, Lukas Greifenstein, Richard Paul Baum

**Affiliations:** 1 CURANOSTICUM Wiesbaden-Frankfurt, Center for Advanced Radiomolecular Precision Oncology, 65191 Wiesbaden, Germany; ghai@curanosticum.de (K.G.); eismant@curanosticum.de (A.E.); parkar@curanosticum.de (T.P.); greifenstein@curanosticum.de (L.G.); baum@curanosticum.de (R.P.B.); 2 Institute of Nuclear Medicine, Università Cattolica del Sacro Cuore, 00168 Rome, Italy; marialucia.calcagni@policlinicogemelli.it (M.L.C.); lucia.leccisotti@policlinicogemelli.it (L.L.); 3 Unit of Nuclear Medicine, Department of Diagnostic Imaging and Radiation Oncology, Fondazione Policlinico Universitario Agostino Gemelli IRCCS, 00168 Rome, Italy; 4 Unità della Fisica per le Scienze della Vita, Fondazione Policlinico Universitario Agostino Gemelli IRCCS, 00168 Rome, Italy; roberto.moretti@guest.policlinicogemelli.it

**Keywords:** Neuroendocrine Neoplasms (NEN), Peptide Receptor Radionuclide Therapy (PRRT), Actinium-225, TANDEM, somatostatin receptor (SSTR) antagonist, DOTA-LM3, safety

## Abstract

Peptide Receptor Radionuclide Therapy (PRRT) offers radiomolecular precision medicine for somatostatin receptor (SSTR)-positive advanced neuroendocrine neoplasms (NEN). In cases resistant to Lutetium-177-labeled DOTATATE or DOTATOC PRRT, alpha-therapy with Actinium-225 labeled with SSTR antagonists like DOTA-LM3 can be a notable therapeutic option. This retrospective study aimed to assess [^225^Ac]Ac-DOTA-LM3 safety in advanced NEN patients (as monotherapy and with Lutetium-177 as TANDEM), survival, and follow-up duration. Thirty-five patients received a total of 57 [^225^Ac]Ac-DOTA-LM3 cycles (March 2022–September 2024): 24 monotherapies and 33 TANDEM therapies. The pancreas was the most common primary site (*n* = 19). PRRT-related toxicity was assessed, focusing on hematological, renal, and hepatic toxicity (Common Terminology Criteria for Adverse Events—CTCAE v5.0). Therapy was generally well tolerated, with mostly mild acute adverse events (primarily nausea, *n* = 8). Some new grade 3/4 long-term adverse events were reported after treatment: anemia grade 3 (*n* = 2), leukocytopenia grade 4 (*n* = 1), absolute neutrophil count reduction grade 3 (*n* = 1), thrombocytopenia grade 3 (*n* = 7), acute myeloid leukemia (*n* = 1), nephrotoxicity grade 3 (*n* = 2), and hepatotoxicity grade 3 (*n* = 2). During follow-up, 13 patients died (survival range 5–30 months); 22 patients were alive (follow-up range 1–18 months). Our retrospective analysis shows that [^225^Ac]Ac-DOTA-LM3 PRRT is relatively safe concerning acute and long-term toxicity and bears promising survival outcomes in patients progressing after [^177^Lu]Lu-DOTATATE or [^177^Lu]Lu-DOTATOC PRRT.

## Simple Summary

Peptide Receptor Radionuclide Therapy (PRRT) using the alpha-emitter Actinium-225 labeled with somatostatin receptor antagonists, such as DOTA-LM3, can be offered to patients with advanced metastatic neuroendocrine neoplasms that are resistant to PRRT with beta-emitters (Lutetium-177 or Yttrium-90). This study retrospectively analyzed the safety and survival outcomes of [^225^Ac]Ac-DOTA-LM3 in 35 patients treated between March 2022 and September 2024. Acute adverse events were mild, with nausea being the most common, and some severe cases of long-term toxicity occurred, including grade 3 anemia, grade 4 leukocytopenia, grade 3 thrombocytopenia, grade 3 nephrotoxicity, and grade 3 hepatotoxicity. Overall, [^225^Ac]Ac-DOTA-LM3 PRRT was well tolerated, showing favorable safety and survival outcomes in patients not responding to previous PRRT options or other conventional therapies.

## 1. Introduction

Neuroendocrine neoplasms (NEN) encompass a variety of tumors commonly located in the gastrointestinal tract, pancreas, and lungs [1]. To standardize histological classification, a universal definition system based on differentiation and proliferative grading was proposed by the World Health Organization (WHO) and the International Agency for Research on Cancer (IARC) [2,3]. Although their incidence and prevalence are increasing, NENs are still considered rare diseases, accounting for about 0.5% of all diagnosed tumors [4]. One of the hallmark features of NENs is the expression of somatostatin receptors (SSTR), which are G-protein–coupled transmembrane receptors involved in cellular proliferation and secretory activity [5], making them ideal candidates for radionuclide-based theranostic approaches that allow the use of both diagnostic and therapeutic nuclides. Peptide Receptor Radionuclide Therapy (PRRT) represents an important example of theranostics, delivering radiation directly to cancer cells at the cellular level, following the first use of receptor imaging and therapy to treat thyroid cancer with radioiodine [6].

The SSTR agonist labeled with the beta-emitter Lutetium-177 ([^177^Lu]Lu-DOTATATE, Lutathera^®^) was the first radiopharmaceutical for PRRT approved by the European Medicines Agency (EMA) in 2017 and by the Food and Drug Administration (FDA) in 2018 for the treatment of SSTR-positive well-differentiated (G1–G2) gastroenteropancreatic NEN [7]. This regulatory approval followed the encouraging results of the first phase-three multicenter clinical trial, NETTER-1, which demonstrated longer progression-free survival (PFS) and a significantly higher response rate than high-dose octreotide long-acting release (LAR) in these patients, with a favorable efficacy–toxicity profile [8].

In metastatic NEN patients progressing after PRRT with beta-emitting nuclides (Lutetium-177 or Yttrium-90) and SSTR agonists (DOTATATE or DOTATOC), two possibilities have become a key area of interest in recent years as part of significant developments in radiotheranostics. The first important novelty is represented by alpha radiation therapy, employing alpha-emitting nuclides such as Lead-212 and particularly Actinium-225, which is characterized by a long half-life (9.9 days) and the emission of four net alpha-particles per decay [9]. Alpha-particles, compared to beta-particles, have higher linear energy transfer (50–230 keV/μm vs. 0.1–1.0 keV/µm), leading to dense radiation damage, a higher probability of DNA double-strand breaks with induced apoptosis, and shorter tissue range penetration (<100 μm vs. 0.05–12 mm) due to higher mass and charge, with reduced damage to surrounding healthy tissues [10,11]. These characteristics make alpha-PRRT particularly indicated for low tumor burden and disseminated disease (e.g., micrometastases). In addition, SSTR antagonists (such as DOTA-JR11, DOTA-LM4, DOTA-LM3) are emerging due to interesting pharmacodynamic features compared to SSTR agonists, including faster association and slower dissociation from SSTR, with consequent low internalization into tumor cells, higher tumor uptake with a higher tumor-to-background ratio, and prolonged tumor retention [12,13,14]. Several studies are currently ongoing to investigate alpha-PRRT as a therapeutic option for PRRT. The study ACTION-1 aims to assess the safety, pharmacokinetics, and recommended phase-three dose of [^225^Ac]Ac-DOTATATE compared to standard of care in patients with inoperable SSTR-positive well-differentiated gastroenteropancreatic NEN who have progressed after PRRT with Lutetium-177-labeled somatostatin analogues [15]. More recently, the results of the phase-three clinical trial NETTER-2 were presented, concerning the use of [^177^Lu]Lu-DOTATATE as first-line therapy in newly diagnosed advanced G2-G3 gastroenteropancreatic NEN patients [16]. Interestingly, the upcoming clinical trial NETTER-3 will evaluate the efficacy and safety of [^177^Lu]Lu-DOTATATE plus octreotide LAR versus octreotide LAR alone in newly diagnosed patients with SSTR-positive well-differentiated G1–G2 (Ki67 < 10%) advanced gastroenteropancreatic NEN with high disease burden [17].

We performed a retrospective analysis after prospective data sampling to evaluate the safety of [^225^Ac]Ac-DOTA-LM3 PRRT (both as monotherapy and as TANDEM-PRRT with Lutetium-177), focusing on acute and long-term adverse events with regard to hematological, renal, and hepatic toxicity. Survival and follow-up duration were also assessed.

## 2. Materials and Methods

We retrospectively enrolled 35 patients with heavily pre-treated metastatic advanced NEN who received at least one cycle of [^225^Ac]Ac-DOTA-LM3 PRRT (both as monotherapy and as TANDEM with Lutetium-177) after the exhaustion of conventional therapeutic options. Treatment was performed at CURANOSTICUM Wiesbaden-Frankfurt, Center for Advanced Radiomolecular Precision Oncology (Wiesbaden, Germany) as compassionate use between March 2022 and September 2024, in compliance with local radiation protection regulations and based on [^68^Ga]Ga-DOTA-LM3 PET/CT findings. Due to the retrospective data acquisition and analysis of this investigation, ethical review and approval were waived. All patients gave their written informed consent to therapy, and all procedures were performed in compliance with the German Medicinal Products Act, AMG §13 2b, the conditions of the Declaration of Helsinki article 37 “Unproven interventions in clinical practice,” and the responsible regulatory body (Government of Hesse).

The eligibility criteria for PRRT were derived from those for PSMA radioligand therapy in patients with metastatic castration-resistant prostate cancer. In short, patients with advanced NEN progressing on standard therapies, ineligible for chemotherapy, or who presented explicit refusal of chemotherapy were included, after a pretherapeutic [^68^Ga]Ga-DOTA-LM3 PET/CT confirming intense and pathological DOTA-LM3 uptake in tumor sites, given a sufficient bone marrow reserve as well as renal and hepatic function. Patients with a negative or weakly positive [^68^Ga]Ga-DOTA-LM3 PET/CT and/or with pre-PRRT severe hematological, renal and/or liver impairment were excluded. The dosing of the radiotherapeutic was patient-specific and considered, among other factors, tumor burden, bone marrow reserve, renal function, and pre-treatments. The total number of cycles was never predetermined but was decided according to the patient’s response after each cycle. TANDEM-PRRT with the combined administration of alpha- and beta-emitting nuclides was indicated in patients with a critically high tumor burden documented at PET/CT, or in patients who had failed or become refractory to monotherapy with beta-PRRT. A two-night hospitalization was required for each cycle of treatment.

The entire cohort (*n* = 35) was studied to assess the occurrence of acute adverse events during or immediately after the administration of [^225^Ac]Ac-DOTA-LM3. Only patients with at least one follow-up at the time of analysis (late September 2024; *n* = 31) were considered to assess long-term safety, survival (calculated using the Kaplan–Meier method), and follow-up duration. Despite our efforts, we were unable to collect follow-up data for 4 patients with NEN, who were considered lost during follow-up due to various reasons, including patients coming from abroad preferring to continue treatment in their home country (*n* = 3) and patients unable to attend follow-up visits because of family or financial constraints (*n* = 1). None of these patients sent any update to our center in terms of clinical or laboratory examinations, therefore it was not possible to include them when evaluating long-term safety. Notably, none of the patients discontinued treatment due to acute or long-term PRRT-related toxicity. Follow-up was conducted both through post-treatment visits in a long-term care setting (all patients were regularly seen and examined starting approximately three months after treatment for as long as they lived) and through visits during subsequent treatment cycles; additionally, contact was maintained between visits. Follow-up included both clinical evaluation and blood sample analyses. In this real-world setting, follow-up intervals were heterogeneous and varied among patients, with some intervals being shorter or longer than others. Long-term safety was analyzed in terms of hematological, renal, and hepatic toxicity. All parameters were prospectively documented in a structured database and retrospectively analyzed. PRRT-related adverse events, both acute and long-term, were graded according to the National Institutes of Health Common Terminology Criteria for Adverse Events (CTCAE v5.0) [18]. Blood samples were obtained to assess hemoglobin, white blood cell count, absolute neutrophil count, platelet count, creatinine, aspartate aminotransferase (AST), alanine aminotransferase (ALT), alkaline phosphatase, and total bilirubin. To evaluate renal function, the estimated glomerular filtration rate, blood urea nitrogen, and electrolyte levels were also considered. In two cases of significant renal impairment, a renal scintigraphy was performed revealing a moderate reduction in renal function. However, these data are not reported and were not included in the analysis, because of the limited number of patients performing renal imaging (*n* = 2) and considering that the main parameter was the creatinine value (available for all patients). Nonetheless, the results derived from renal imaging were taken into account for clinical purposes.

Patients received [^225^Ac]Ac-DOTA-LM3 PRRT based on [^68^Ga]Ga-DOTA-LM3 findings. In specific cases, patients underwent other imaging modalities to provide a better assessment of the disease (e.g., [^18^F]FDG PET/CT, contrast-enhanced CT scans, or MRI), but results were not used as eligibility criteria for PRRT, nor were they included in the protocol for selection.

Premedication to avoid nausea and emesis was administered before each cycle of PRRT through intravenous administration of dexamethasone (8 mg) and granisetron (3 mg) 15–20 min before infusion; in case of nausea after the infusion of the radiotherapeutic and removal of the intravenous catheter, ondansetron (8 mg) was orally administered. Moreover, hydration was provided with saline solution (1000 mL) with instructions to drink mineral water; renal protection was managed via pump intravenous infusion of para-aminohippuric acid (100 mL) for the following 70 min. [^225^Ac]Ac-DOTA-LM3 was administered through a slow intravenous infusion (10–15 min or longer, depending on the patient’s well-being) to control possible SSTR overstimulation and acute side effects, and meanwhile blood pressure and pulse were monitored. In patients undergoing TANDEM-PRRT, a concept that implies the administration of two different radionuclides, one alpha-emitter (in this case Actinium-225) and one be-ta-emitter (in this case Lutetium-177), [^225^Ac]Ac-DOTA-LM3 and [^177^Lu]Lu-DOTA-LM3 were administered on the same day concomitantly, or on two consecutive days; in these patients, SPECT/CT was performed the day after the injection of the Luteti-um-177-labeled radiotherapeutic molecule to assess the intensity and distribution of uptake.

## 3. Results

### 3.1. Patient Cohort and PRRT Data

Our cohort included 35 heavily pre-treated metastatic NEN patients (24 males and 11 females; age range 36–86 years at the time of the first [^225^Ac]Ac-DOTA-LM3 PRRT). The most common primary site was the pancreas (*n* = 19), followed by the small bowel (*n* = 10) and rectum (*n* = 2); the cohort also included esthesioneuroblastoma (*n* = 1), pheochromocytoma (*n* = 1), ovary (*n* = 1), and NEN of unknown primary (*n* = 1). Patients had been previously treated as follows: PRRT with SSTR agonists labeled with beta-emitters (91.4%); therapy with somatostatin “cold” analogues (85.7%); surgery of the primary tumor, lymph nodes or distant metastases, or interventional procedures (e.g., trans-arterial chemoembolization) (80%); chemotherapy (51.4%); therapy with Everolimus (34.3%); external beam radiation therapy of the primary tumor, lymph nodes or distant metastases (28.6%); PRRT with SSTR antagonists labeled with beta-emitters (28.6%); therapy with tyrosine-kinase inhibitors (22.8%); other therapies (e.g., Nivolumab) (14.3%); or PRRT with SSTR agonists labeled with alpha-emitters (11.4%). A table focusing on patients’ characteristics can be found in the Supplementary Material (Table S1).

**Figure 1:**
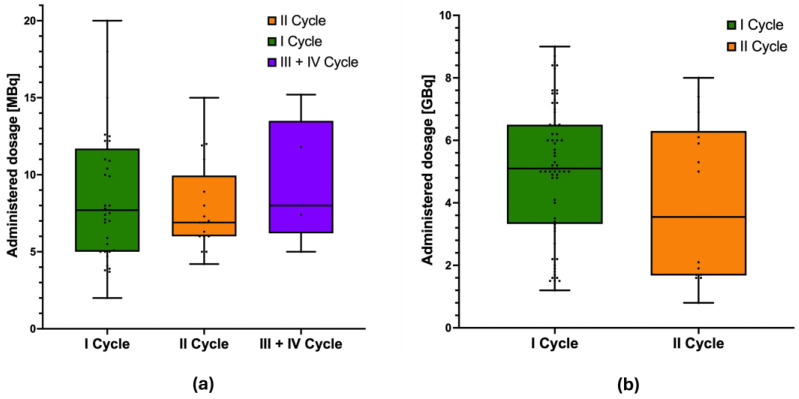
Boxplots of the administered ^225^Ac (**a**) and ^177^Lu (**b**) dosage according to the cycle.

Over a period of 30 months, 57 [^225^Ac]Ac-DOTA-LM3 cycles were administered (24 Actinium-225 monotherapies and 33 TANDEM-PRRT as [^225^Ac]Ac-/[^177^Lu]Lu-DOTA-LM3). At the time of analysis, 18 patients had received one cycle, either monotherapy or TANDEM, 13 patients had received two cycles, and 4 patients had received three or more cycles (*n* = 3, three cycles; *n* = 1, four cycles). Figure 1 shows the boxplots of the Actinium-225 (a) and Lutetium-177 (b) dosage administered in the first, second, and subsequent cycles, respectively.

PRRT was generally well tolerated, with only a few acute adverse events of mild severity (all grade 1 according to CTCAE v.5.0). The most common acute adverse event was nausea (*n* = 8, 22.8%), followed by emesis (*n* = 7, 20%), flushing (*n* = 7, 20%), flare pain (*n* = 6, 17.1%), diarrhea (*n* = 3, 8.6%), fatigue (*n* = 2, 5.7%), and hypertension (*n* = 1, 2.8%). All reported acute adverse events were either self-limiting or, when needed, managed with routine medications (analgesics and antiemetics); management of flushing did not require medical intervention; the single case of mild hypertension did not require medical intervention, and the patient was monitored to evaluate the trend of blood pressure in the hours following injection.

### 3.2. Survival Analyses

**Figure 2:**
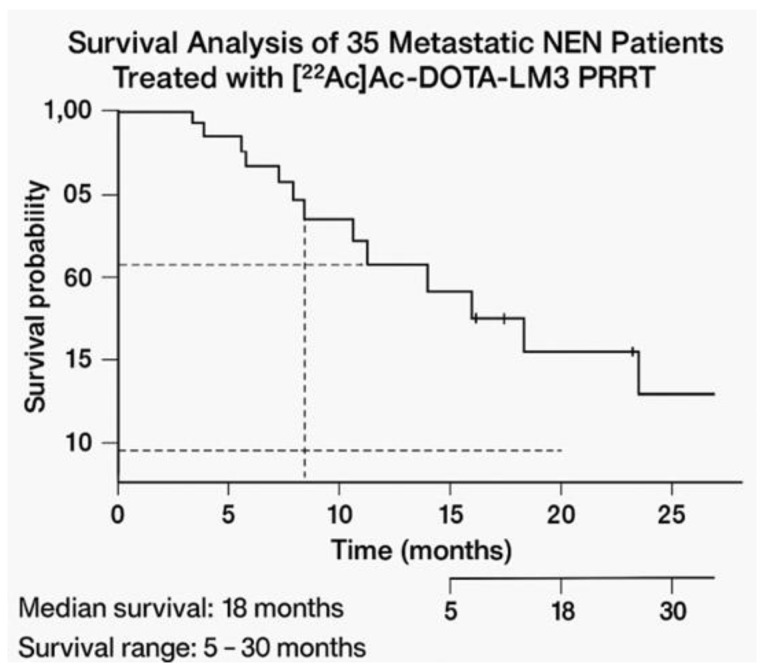
Kaplan–Meier curve.

Thirteen patients passed away (survival between 5 and 30 months; median: 18 months) and 22 patients were alive (follow-up duration range 1–18 months). The Kaplan–Meier curve is shown in Figure 2. The overall follow-up duration range, obtained considering all patients, was 1–30 months (median: 11 months).

### 3.3. Hematological Toxicity

Hematological long-term toxicity was assessed in 31 patients, considering hemoglobin levels, white blood cell count, and platelet count. Absolute neutrophil count was also monitored. The management of severe long-term hematological toxicity was supportive, including blood transfusions and administration of growth factors. The distribution of patients before and after treatment according to these parameters is displayed in Table 1.

**Figure 3:**
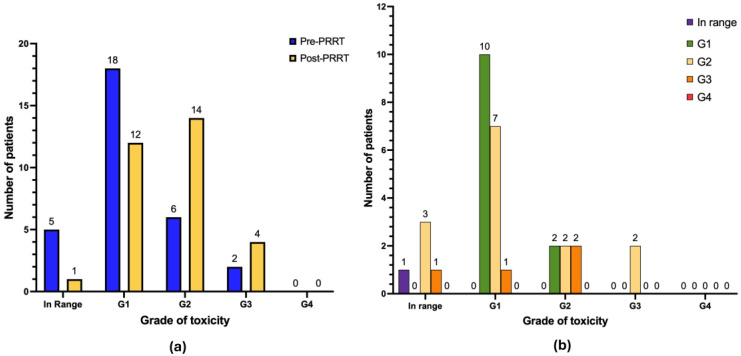
Graphical representation of hematological long-term toxicity regarding anemia (*n* = 31 patients). (**a**) Absolute number of patients in pre-PRRT (blue) and post-PRRT (yellow) settings, according to normal hemoglobin values or different grades of anemia. (**b**) Distribution of patients showing the progression or regression of anemia severity. Legend (CTCAE v5.0): in range: hemoglobin (Hb) > lower limit of normal (LLN); anemia G1 (Hb < LLN to 10 g/dL); anemia G2 (Hb 8–10 g/dL); anemia G3 (Hb < 8 g/dL), transfusion indicated; anemia G4 (life-threatening consequences), urgent intervention indicated.

With regard to anemia (Figure 3b), after treatment 3 patients manifested de novo anemia G2 (9.7%) and 1 patient manifested de novo anemia G3 (3.2%). Seven patients progressed from anemia G1 to G2 (22.6%), 1 patient progressed from anemia G1 to G3 (3.2%) and 2 patients progressed from anemia G2 to G3 (6.4%). No cases of anemia G4 were observed. The remaining patients of the cohort either continued to have normal values of hemoglobin (*n* = 1), or the same grade of anemia as reported at baseline pre-PRRT (*n* = 10 anemia G1; *n* = 2 anemia G2), or their hemoglobin values improved during follow-up (*n* = 4).

**Figure 4:**
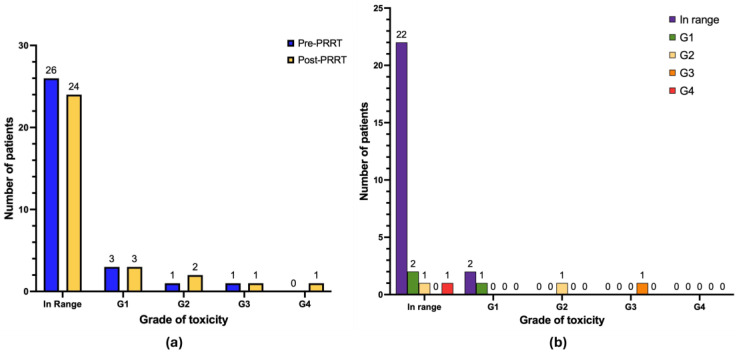
Graphical representation of hematological long-term toxicity regarding leukocytopenia (*n* = 31 patients). (**a**) Absolute number of patients in pre-PRRT (blue) and post-PRRT (yellow) settings, according to normal white blood cell values or different grades of leukocytopenia. (**b**) Distribution of patients showing the progression or regression of leukocytopenia severity; only patients with available paired pre- and post-PRRT data are included. Legend (CTCAE v5.0): in range: white blood cell (WBC) > lower limit of normal (LLN); leukocytopenia G1 (WBC < LLN to 3000/mm^3^); leukocytopenia G2 (WBC 2000–3000/mm^3^); leukocytopenia G3 (WBC 1000–2000/mm^3^); leukocytopenia G4 (WBC < 1000/mm^3^).

When considering leukocytopenia (Figure 4b), after treatment 2 patients developed de novo leukocytopenia G1 (6.4%), 1 patient developed de novo leukocytopenia G2 (3.2%), and 1 patient developed de novo leukocytopenia G4 (3.2%). No cases of worsening of baseline leukocytopenia were recorded. The remaining patients in the cohort either continued to have a normal white blood cell count (*n* = 22), maintained the same grade of leukocytopenia as reported at baseline pre-PRRT (*n* =1 leukocytopenia G1; *n* = 1 leukocytopenia G2; *n* = 1 leukocytopenia G3), or their white blood cell count improved during follow-up (*n* = 2). We documented one case of neutropenia G3 (absolute neutrophil count < 1000–500/mm^3^) in a patient with metastatic small intestine NEN previously treated with chemotherapy and beta-PRRT; it was observed three weeks after TANDEM therapy and lasted for about three months before completely resolving. A patient with metastatic pancreatic NEN G3, previously treated with surgery, trans-arterial chemoembolization of hepatic metastases, therapy with somatostatin “cold” analogue, chemotherapy, therapy with Everolimus and beta-PRRT, was diagnosed with acute myeloid leukemia twelve years after the first [^90^Y]Y-DOTATOC PRRT and three months after DOTA-LM3 TANDEM-PRRT. Therapy-related myeloid neoplasms are a well-known infrequent but serious complication in this setting [19]. The patient was unfortunately unsuccessfully treated with chemotherapy and supportive therapy. It is not possible to establish a cause-effect relationship between the DOTA-LM3 TANDEM-PRRT and the diagnosis of myeloid leukemia (also given the short interval between the two events), but previous treatments-particularly prior chemotherapy and [^90^Y]Y-DOTATOC PRRT-must certainly be taken into account considering their potential pro-oncogenic effect.

**Figure 5:**
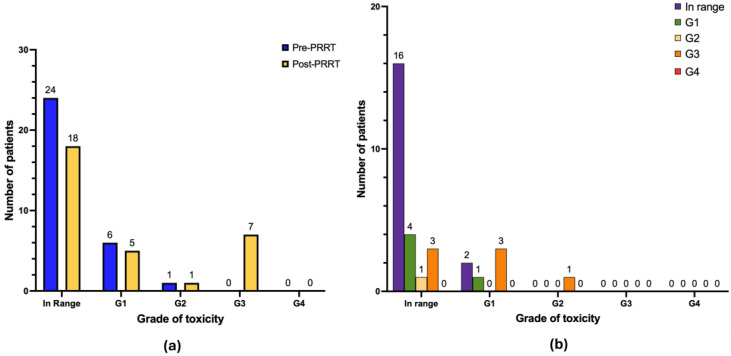
Graphical representation of hematological long-term toxicity regarding thrombocytopenia (*n* = 31 patients). (**a**) Absolute number of patients in pre-PRRT (blue) and post-PRRT (yellow) settings, according to normal platelet values or different grades of thrombocytopenia. (**b**) Distribution of patients showing the progression or regression of thrombocytopenia severity; only patients with available paired pre- and post-PRRT data are included. Legend (CTCAE v5.0): in range: platelet (PTL) ≥ lower limit of normal (LLN); thrombocytopenia G1 (PTL < LLN to 75,000/mm^3^); thrombocytopenia G2 (PTL 50,000–75,000/mm^3^); thrombocytopenia G3 (PTL 25,000–50,000/mm^3^); thrombocytopenia G4 (PTL < 25,000/mm^3^).

With regard to thrombocytopenia (Figure 5b), after treatment 4 patients developed de novo thrombocytopenia G1 (12.9%), 1 patient developed de novo thrombocytopenia G2 (3.2%), and 3 patients developed de novo thrombocytopenia G3 (9.7%). Three patients progressed from thrombocytopenia G1 to G3 (9.7%) and 1 patient progressed from thrombocytopenia G2 to G3 (3.2%). No cases of thrombocytopenia G4 were observed. The remaining patients in the cohort either continued to have normal platelet count (*n* = 16), maintained the same grade of thrombocytopenia as at baseline (*n* = 1 thrombocytopenia G1), or showed improved platelet count during follow-up (*n* = 2).

### 3.4. Nephrotoxicity and Hepatotoxicity

Long-term nephrotoxicity and hepatotoxicity were assessed in 31 patients, considering values of creatinine, transaminases (AST and ALT), alkaline phosphatase, and total bilirubin, respectively. The distribution of patients before and after treatment according to these parameters is displayed in Table 2.

**Figure 6:**
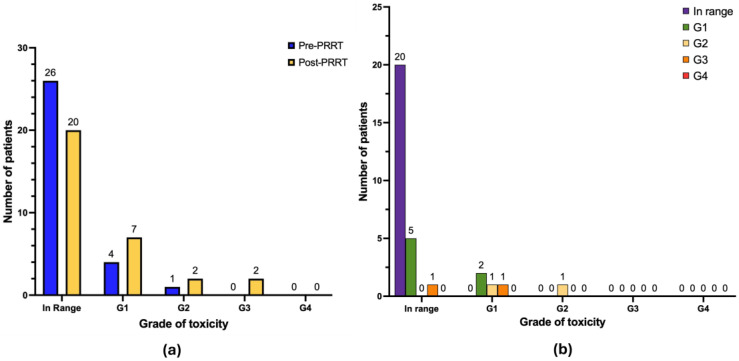
Graphical representation of long-term nephrotoxicity (*n* = 31 patients). (**a**) Absolute number of patients in pre-PRRT (blue) and post-PRRT (yellow) settings, according to normal serum creatinine values or different grades of nephrotoxicity. (**b**) Distribution of patients showing the progression or regression of nephrotoxicity severity; only patients with available paired pre- and post-PRRT data are included. Legend (CTCAE v5.0): in range: serum creatinine < upper limit of normal (ULN); renal insufficiency G1 (serum creatinine 1.0–1.5 × ULN); renal insufficiency G2 (1.5–3.0 × ULN); renal insufficiency G3 (3.0–6.0 × ULN); renal insufficiency G4 (>6.0 × ULN).

With regard to nephrotoxicity (Figure 6b), after treatment 20 patients had no renal impairment (64.5%), 5 patients developed de novo renal impairment G1 (16.1%), and 1 patient developed de novo renal impairment G3 (3.2%; in this case, considering the glomerular filtration of radiotherapeutics, a renal biopsy was performed revealing tubular damage without glomerular disturbances, thus excluding PRRT-related toxicity). One patient progressed from renal impairment G1 to G2 (3.2%), and 1 patient progressed from renal impairment G1 to G3 (3.2%; this patient had a personal history of neurogenic bladder with recurrent urinary retention and infections). No cases of renal impairment G4 were observed. Other patients in our cohort continued to exhibit the same degree of renal impairment as documented at baseline pre-PRRT (*n* = 2 renal impairment G1; *n* = 1 renal impairment G2).

**Figure 7:**
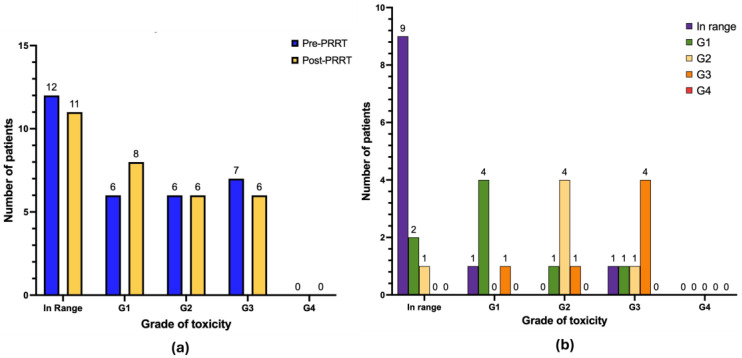
Graphical representation of long-term hepatotoxicity (*n* =3 1 patients). (**a**) Absolute number of patients in pre-PRRT (blue) and post-PRRT (yellow) settings, according to normal liver function values or different grades of hepatotoxicity. (**b**) Distribution of patients showing progression or regression of hepatotoxicity severity; only patients with available paired data are included. Legend (CTCAE v5.0): G0 (in range): AST/ALT/ALP/bilirubin < ULN; G1: AST/ALT > ULN–3.0 × ULN, ALP > ULN–2.5 × ULN, bilirubin > ULN–1.5 × ULN; G2: AST/ALT > 3.0–5.0 × ULN, ALP > 2.5–5.0 × ULN, bilirubin > 1.5–3.0 × ULN; G3: AST/ALT > 5.0–20.0 × ULN, ALP > 5.0–20.0 × ULN, bilirubin > 3.0–10.0 × ULN; G4: AST/ALT > 20.0 × ULN, ALP > 20.0 × ULN, bilirubin > 10.0 × ULN.

With regard to hepatotoxicity (Figure 7b), after treatment 11 patients had no liver toxicity (35.5%), 2 patients developed de novo liver impairment G1 (6.4%), and 1 patient developed de novo liver impairment G2 (3.2%). One patient progressed from liver impairment G1 to G3 (3.2%), and another patient progressed from liver impairment G2 to G3 (3.2%). No cases of liver impairment G4 were observed. The remaining patients in our cohort either continued to exhibit the same degree of liver impairment as documented at baseline pre-PRRT (*n* = 4 liver impairment G1; *n* = 4 liver impairment G2; *n* = 4 liver impairment G3) or showed improvement in hepatic function during follow-up (*n* = 5).

**Figure 8:**
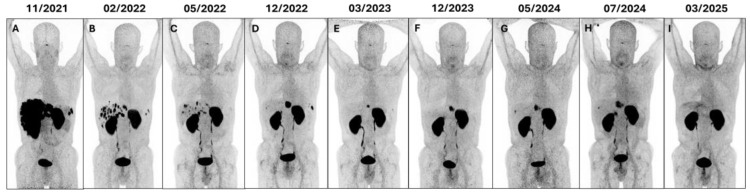
MIP sequence of [^68^Ga]Ga-DOTA-LM3 PET/CT scans of a 52-year-old patient diagnosed in 2014 with well-differentiated, functioning NEN of the pancreas G3 (Ki67 52%). He underwent pancreaticoduodenectomy and, due to hepatic and peritoneal lesions, received chemotherapy (2014), liver metastases resection (2018), 4 cycles of [^177^Lu]Lu-Oxodotreotide (Lutathera^®^, 2020), Sunitinib, Everolimus, Pembrolizumab, and somatostatin analogues (2021). Restaging with Gallium-68-labeled SSTR antagonist PET/CT ((**A**); 11/2021) showed massive progression with innumerable liver metastases and increased chromogranin A (>2000 µg/L), prompting two cycles of [^177^Lu]Lu-DOTA-LM3 PRRT (11/2021–02/2022), achieving partial remission (**B**,**C**). He also received immunotherapy with Nivolumab. Peritoneal recurrence in the upper abdomen led to two more cycles of [^177^Lu]Lu-DOTA-LM3 PRRT (12/2022–03/2023), resulting in minimal residual disease at [^68^Ga]Ga-DOTA-LM3 PET/CT (**D**,**E**). Subsequent [^68^Ga]Ga-DOTA-LM3 PET/CT scans (**F**–**H**) documented abdominal progression with increased chromogranin A (>8000 µg/L); therefore, he received [^177^Lu]Lu-DOTA-LM3 (08/2024) and [^225^Ac]Ac-DOTA-LM3 TANDEM-PRRT (10/2024), which was well tolerated. The patient benefited from PRRT, becoming physically active without any toxicity during follow-up; this allowed him to successfully undergo hepatic and diaphragmatic surgery in late 2024. [^68^Ga]Ga-DOTA-LM3 PET/CT in March 2025 (**I**) demonstrated complete remission, particularly in the para-gastric region and liver. As of April 2025, the patient is alive and in excellent clinical condition (having recently won a pickleball competition), 6 months after TANDEM-PRRT and 11 years after diagnosis.

An example of a patient with pancreatic NEN treated with PRRT who experienced no hematological, renal, or hepatic toxicity and achieved complete remission of disease is shown in Figure 8.

## 4. Discussion

The results of this retrospective analysis with prospective documentation indicate that [^225^Ac]Ac-DOTA-LM3 PRRT is overall safe in heavily pre-treated NEN patients who have progressed after conventional treatments. Only a few transient and mild acute adverse events were observed, primarily nausea, which were usually self-limiting or otherwise manageable with routine medications (e.g., antiemetics). Severe hematological, renal, and hepatic long-term toxicities were rare. After treatment, we documented the following severe (G3/G4) long-term adverse events: *n* = 2 anemia G3, *n* = 1 leukocytopenia G4, *n* = 1 absolute neutrophil count reduction G3, *n* = 1 acute myeloid leukemia in a patient previously treated with chemotherapy and [^90^Y]Y-DOTATOC PRRT, *n* = 7 thrombocytopenia G3, *n* = 2 nephrotoxicity G3 (demonstrated not to be PRRT-related), *n* = 2 hepatotoxicity G3 (in patients with liver metastases). When analyzing long-term toxicities, additional factors must be considered alongside the potential direct effect of the radioisotope, particularly previous cytotoxic treatments, pre-existing conditions, and localization of metastases (mainly bone marrow and liver). Furthermore, this study demonstrated promising survival outcomes with [^225^Ac]Ac-DOTA-LM3 PRRT in heavily pre-treated patients who had failed all available treatment options, including PRRT with beta-emitting SSTR agonists. In our cohort, survival reached up to 30 months, highlighting its potential survival benefit in this challenging clinical setting.

Patients suffering from metastatic progressive NEN which is resistant to conventional treatments, although representing only a subset of those diagnosed with this condition, present a significant therapeutic challenge in oncology. The phase-three clinical trial NETTER-1 has been one of the most important milestones in recent years, establishing a validated therapeutic option for gastroenteropancreatic G1–G2 NEN patients who otherwise have limited treatment options after progression on first-line somatostatin analogue therapy [8]. This trial randomized 229 patients to receive either [^177^Lu]Lu-DOTATATE PRRT (116 patients; four intravenous infusions of 7.4 GBq each, every 8 weeks) plus best supportive care, including octreotide LAR (30 mg) administered intramuscularly, or octreotide LAR alone (60 mg, 113 patients) administered intramuscularly every 4 weeks. It showed that the estimated rate of PFS (primary endpoint) at month 20 was 65.2% in the [^177^Lu]Lu-DOTATATE group and 10.8% in the control group. Secondary endpoints included response rate (18% in the [^177^Lu]Lu-DOTATATE group vs. 3% in the control group), overall survival (OS; 14 deaths in the [^177^Lu]Lu-DOTATATE group vs. 26 in the control group), and safety (no evidence of nephrotoxicity was reported and only a few cases of G3/G4 hematological adverse events were observed in the [^177^Lu]Lu-DOTATATE group). Importantly, given the deterioration in quality of life associated with advanced NEN (especially when functional), the impact of [^177^Lu]Lu-DOTATATE PRRT on quality of life was also assessed, demonstrating a significant benefit for patients with progressive NEN compared with high-dose octreotide [20].

Subsequently, the need to expand the therapeutic field to NEN patients with other characteristics emerged; the phase-three clinical trial NETTER-2 was recently conducted to determine whether [^177^Lu]Lu-DOTATATE in combination with long-acting octreotide could prolong PFS in 226 patients with high-grade (G2–G3) gastroenteropancreatic NEN when given as first-line treatment (151 patients, both somatostatin analogue-naive and previously treated with somatostatin analogues without progression) compared to high-dose (60 mg) long-acting octreotide (75 patients) [21]. The results were recently presented and were very encouraging: median PFS was 22.8 months in the [^177^Lu]Lu-DOTATATE group vs. 8.5 months in the control group; adverse events of any grade occurred in 93% of patients in the [^177^Lu]Lu-DOTATATE group vs. 95% in the control group; and there were no study drug-related deaths. Therefore, [^177^Lu]Lu-DOTATATE should be considered a new standard of care in first-line therapy in this patient population [16]. Further populations will be studied to assess whether [^177^Lu]Lu-DOTATATE PRRT can help as first-line therapy in newly diagnosed G1–G2 NEN patients (NETTER-3 trial) [17]. In addition, there remains a need for standardized investigations and systematic evaluation of PRRT in non-gastroenteropancreatic NEN.

Unfortunately, there are still patients resistant to beta-PRRT with SSTR analogues. In countries where Actinium-225 is available (such as Germany), alpha-PRRT can be proposed to these patients by labeling the SSTR agonist with Actinium-225, especially to treat micrometastatic disease due to the physical characteristics of alpha particles compared to beta particles, including higher linear energy transfer and shorter tissue penetration range [10,11]. The most appropriate single cycle, cumulative doses, and long-term safety of [^225^Ac]Ac-DOTATOC have been studied in these patients [22]. Other authors have studied the efficacy and safety of [^225^Ac]Ac-DOTATATE in patients with NEN, showing good results in terms of molecular imaging-based response and a disease control rate of 80%, without severe toxicities or treatment-related deaths [23].

Another growing field of interest in overcoming the therapeutic limitations of PRRT with SSTR analogues derives from the study of the SSTR, which, being a G-protein-coupled receptor, exists in different conformations (active and inactive). Agonists stimulate the SSTR as the natural ligand somatostatin does, causing internalization of the receptor–ligand complex, which is needed to reduce background when performing imaging. However, as early as 2006, Ginj et al. demonstrated in vitro that SSTR express more binding sites for antagonists than for agonists, probably because of the ability to recruit inactive receptors to the cell surface [24]. This was the first demonstration that internalization was not mandatory and that radiolabeled SSTR antagonists may perform better than agonists despite the lack of internalization. Therefore, the application of SSTR antagonists for PRRT (DOTA-JR11, DOTA-LM4, DOTA-LM3, etc.), labeled with either beta- or alpha-emitters, represents an extremely important perspective in this field [25,26]. A pilot study of PRRT with SSTR antagonists was conducted in 2014 by Wild et al.; four patients with advanced NEN were treated with [^177^Lu]Lu-DOTA-JR11, which showed a 1.7–10.6 times higher tumor dose than [^177^Lu]Lu-DOTATATE and a 1.1–7.2 times higher tumor-to-kidney and tumor-to-bone marrow dose ratio [27]. A few years later, the first-in-human study to assess the safety, dosimetry, and efficacy of [^177^Lu]Lu-DOTA-LM3 was performed by Baum et al. in 51 patients with metastatic NEN. The authors demonstrated a favorable biodistribution and higher uptake with longer effective half-life of [^177^Lu]Lu-DOTA-LM3 compared to [^177^Lu]Lu-DOTATOC in the whole body, kidneys, spleen, and metastases, resulting in higher mean absorbed organ and tumor doses, without any serious acute adverse effects (especially no nephrotoxicity) and with a disease control rate of 85.1% [13]. A preclinical evaluation of Actinium-225-labeled DOTA-JR11 compared to Lutetium-177-labeled DOTA-JR11 was recently published [28], as well as initial cases of PRRT with [^225^Ac]Ac-DOTA-LM3, also as TANDEM combining [^225^Ac]Ac-DOTA-LM3 with [^177^Lu]Lu-DOTA-LM3 [29,30].

A potential paradigm shift in PRRT is Terbium-161, which decays emitting not only beta particles, but also conversion and Auger electrons, which are believed to be effective in killing single cancer cells [31]. Both SSTR agonists and antagonists have been studied in combination with Terbium-161 for PRRT in NEN patients, and it may represent a new feasible therapeutic option [32,33]. Along with Terbium-161, Lead-212, a beta-emitter with alpha-emitting daughters, is also under investigation, and clinical applications have been explored [34]. For instance, a phase-one first-in-human dose-escalation trial evaluated the use of [^212^Pb]Pb-DOTAMTATE (a bifunctional metal chelator [DOTAM] and SSTR agonist [TATE]) in twenty patients with NEN and found it to be well tolerated [35].

[^225^Ac]Ac-DOTA-LM3 PRRT can be administered as monotherapy or in TANDEM with [^177^Lu]Lu-DOTA-LM3, integrating the complementary physical properties of both nuclides, with significant clinical outcomes. Further studies are warranted to clarify whether TANDEM-PRRT should be administered as initial therapy or as second-line therapy in NEN patients progressing after beta-PRRT. Additionally, to improve therapeutic outcomes in patients resistant to internal radiation, PRRT can be administered concurrently with other treatments considered standard pillars in cancer therapy, in the form of COMBO-PRRT [36]. Several ongoing trials are currently investigating the use of [^177^Lu]Lu-DOTATATE (Lutathera^®^) combined with other agents, for example, PARP inhibitors, tyrosine-kinase inhibitors, mTOR inhibitors, and immunotherapy [37]. Not surprisingly, the growing potential of combo-PRRT is also being explored with alpha-emitters, for example, by concomitantly administering a chemotherapeutic agent as a radiosensitizer [38].

## 5. Conclusions

Alpha-PRRT with [^225^Ac]Ac-DOTA-LM3 (either as monotherapy or in TANDEM) in NEN patients who have failed previous treatments, including beta-PRRT and chemotherapy, is safe overall and demonstrates promising survival outcomes. This emerging therapeutic approach highlights a thriving area of clinical and research innovation in radiotheranostics, paving the way toward personalized medicine.

## Figures and Tables

**Table 1:** Distribution of patients before and after PRRT considering anemia, leukocytopenia and thrombocytopenia.

	Before Treatment		After Treatment	
**Anemia (Grading)**	**Number (*n*)**	**Percent (%)**	**Number (*n*)**	**Percent (%)**
G0	5	16.1	1	3.2
G1	18	58.0	12	38.7
G2	6	19.3	14	45.1
G3	2	6.4	4	12.9
G4	0	0.0	0	0.0
**Leukocytopenia (Grading)**	**Number (*n*)**	**Percent (%)**	**Number (*n*)**	**Percent (%)**
G0	26	83.9	24	77.4
G1	3	9.7	3	9.7
G2	1	3.2	2	6.4
G3	1	3.2	1	3.2
G4	0	0.0	1	3.2
**Thrombocytopenia (Grading)**	**Number (*n*)**	**Percent (%)**	**Number (*n*)**	**Percent (%)**
G0	24	77.4	18	58
G1	6	19.3	5	16.1
G2	1	3.2	1	3.2
G3	0	0.0	7	22.6
G4	0	0.0	0	0.0

**Table 2:** Distribution of patients before and after PRRT according to renal and hepatic function.

	Before Treatment		After Treatment	
**Renal Function (Grading)**	**Number (*n*)**	**Percent (%)**	**Number (*n*)**	**Percent (%)**
G0	26	83.9	20	64.5
G1	4	12.9	7	22.6
G2	1	3.2	2	6.4
G3	0	0.0	2	6.4
G4	0	0.0	0	0.0
**Hepatic Function (Grading)**	**Number (*n*)**	**Percent (%)**	**Number (*n*)**	**Percent (%)**
G0	12	38.7	11	35.5
G1	6	19.3	8	25.8
G2	6	19.3	6	19.3
G3	7	22.6	6	19.3
G4	0	0.0	0	0.0

## Data Availability

The raw data supporting the conclusions of this article will be made available by the authors upon request.

## Supplementary Materials

The following supporting information can be downloaded at: https://www.mdpi.com/article/10.3390/cancers17183070/s1, Table S1. Patients’ characteristics.
