# Supplementary material for: Peptide Receptor Radionuclide Therapy (PRRT) Using Actinium-225- and Ac-225/Lutetium-177-Labeled (TANDEM) Somatostatin Receptor Antagonist DOTA-LM3 in Patients with Neuroendocrine Neoplasm: A Retrospective Study Concerning Safety and Survival
Source: Cancers (Basel). 2025 Sep 19;17(18):3070. doi: 10.3390/cancers17183070 (PMC12468063; doi:10.3390/cancers17183070)
Supplement: Supplementary file 1 [file cancers-17-03070-s001.zip › cancers-3760530-supplementary.pdf]

Table S1. Patients' characteristics.

**Patient number**	**Gender**	**Age***	**NEN site**	**Tumor grade**	**Ki67 (%)**	**Previous treatments**	**Alpha-PRRT (n. cycles)**	**TANDEM-PRRT (n. cycles)**	**Main tumor sites**	**Toxicity outcomes (long-term)**
**1**	M	40	Pancreas	G2	10	Surgery, liver TACE, SST analogue, chemotherapy, beta-PRRT with SSTR agonist, beta-PRRT with SSTR antagonist, TKi, Everolimus	2	0	Bone, liver	Anemia G2
**2**	M	57	Pancreas	G3	25	Surgery, SST analogue, radiotherapy, beta-PRRT with SSTR agonist	1	1	Bone, liver	Anemia G3, leukocytopenia G2
**3**	M	38	Small intestine	G3	50	Chemotherapy, radiotherapy, beta-PRRT with SSTR agonist, beta-PRRT with SSTR antagonist, Everolimus	0	1	Bone, liver, LNM	Thrombocytopenia G3. Transient ANC reduction G3
**4**	M	36	Pancreas	G2	8	Liver TACE, radiotherapy, beta-PRRT with SSTR agonist, alpha-PRRT with SSTR agonist, TKi, Everolimus	0	2	Bone, liver	Thrombocytopenia G1, nephrotoxicity G1, hepatotoxicity G3
**5**	M	57	Pancreas	G3	21	Surgery, liver TACE, SST analogue, chemotherapy, beta-PRRT with SSTR agonist, alpha-PRRT with SSTR agonist, Everolimus	0	1	Bone, liver	AML (three months after DOTA-LM3 TANDEM-PRRT; twelve years after [^{90}Y]Y-DOTATOC PRRT)
**6**	F	61	Pancreas	G2	12	Surgery, SST analogue, beta-PRRT with SSTR agonist	1	0	Bone, liver	Nephrotoxicity G3
**7**	M	46	Pancreas	G2	8	Surgery, SST analogue, radiotherapy	2	0	Liver, LNM	Nephrotoxicity G3
**8**	F	60	Small intestine	G2	10	Surgery, SST analogue, chemotherapy, beta-PRRT with SSTR agonist, interferon	1	0	LNM	Lost during follow-up
**9**	M	52	Pancreas	G2	14	SST analogue, beta-PRRT with SSTR agonist, beta-PRRT with SSTR antagonist , Everolimus	1	0	Bone, liver	Anemia G2, leukocytopenia G1

**10**	M	60	Rectum	G2	12	Surgery, SST analogue, beta-PRRT with SSTR agonist, Nivolumab	2	0	Bone, liver, LNM	Nephrotoxicity G1
**11**	F	51	Pancreas	G2	10	Surgery, SST analogue, chemotherapy, beta-PRRT with SSTR agonist	1	2	LNM	Hepatotoxicity G1
**12**	M	60	Pancreas	G3	47	Surgery, SST analogue, chemotherapy, beta-PRRT with SSTR agonist	0	2	Bone, liver, LNM	Thrombocytopenia G1
**13**	M	65	Pancreas	G3	21	SST analogue, chemotherapy, beta-PRRT with SSTR agonist, Nivolumab	0	1	Bone, liver, LNM	Anemia G2, thrombocytopenia G1, hepatotoxicity G2
**14**	F	42	Small intestine	G3	30	Surgery, SST analogue, chemotherapy, beta-PRRT with SSTR agonist, TKi, Everolimus	1	1	Bone, liver	Anemia G2, nephrotoxicity G1, hepatotoxicity G3
**15**	M	67	Small intestine	G1	2	Surgery, SST analogue, beta-PRRT with SSTR agonist	1	0	Bone, liver	Anemia G2
**16**	M	43	Pheochromocytoma	N.r.	N.r.	Surgery, beta-PRRT with SSTR agonist, [^{131}I]I-MIBG radioligand therapy	1	1	Bone	Anemia G3, thrombocytopenia G3
**17**	F	58	Pancreas	G3	30	Surgery, SST analogue, chemotherapy, beta-PRRT with SSTR agonist, Everolimus	1	3	Bone, liver, LNM	Anemia G2, thrombocytopenia G3
**18**	M	44	Rectum	G3	35	SST analogue, chemotherapy, beta-PRRT with SSTR agonist	1	2	Bone, liver, LNM	Anemia G2
**19**	F	56	NEN of unknown primary	G2	12	SST analogue, radiotherapy, beta-PRRT with SSTR agonist	0	1	Bone, liver, LNM	Anemia G3, thrombocytopenia G3
**20**	M	44	Pancreas	G2	16	Surgery, liver TACE, SST analogue, chemotherapy, beta-PRRT with SSTR agonist, alpha-PRRT with SSTR agonist, TKi, Everolimus	1	0	Bone, liver	Nephrotoxicity G1

**21**	M	49	Pancreas	G2	14	Surgery, SST analogue, chemotherapy, beta-PRRT with SSTR agonist, beta-PRRT with SSTR antagonist	0	1	Bone, liver, LNM	None
**22**	M	55	Small intestine	G2	8	Surgery, liver TACE, SST analogue, beta-PRRT with SSTR agonist, beta-PRRT with SSTR antagonist, Everolimus	0	2	Bone, liver	Thrombocytopenia G1
**23**	F	44	Pancreas	G3	30	Surgery, liver TACE, SST analogue, beta-PRRT with SSTR agonist, alpha-PRRT with SSTR agonist	0	2	Bone, liver, LNM	Thrombocytopenia G2
**24**	F	57	Small intestine	G1	2	Surgery, SST analogue, radiotherapy, beta-PRRT with SSTR agonist, TKi	1	2	Bone, liver	Anemia G3, leukocytopenia G1, thrombocytopenia G3
**25**	M	86	Small intestine	G2	8	Surgery, SST analogue, beta-PRRT with SSTR agonist	1	0	Liver	Lost during follow-up
**26**	F	45	Esthesio-neuroblastoma	G3	45	Surgery, chemotherapy, radiotherapy, beta-PRRT with SSTR agonist	0	2	Bone, LNM, soft tissues	Anemia G2, hepatotoxicity G1
**27**	M	78	Pancreas	G2	14	Surgery, SST analogue, beta-PRRT with SSTR agonist, beta-PRRT with SSTR antagonist	1	0	Liver	Anemia G2, nephrotoxicity G1
**28**	M	54	Pancreas	G2	10	Beta-PRRT with SSTR agonist, beta-PRRT with SSTR antagonist	0	2	Bone, liver	Nephrotoxicity G2
**29**	M	58	Pancreas	G3	45	SST analogue, chemotherapy, beta-PRRT with SSTR agonist, beta-PRRT with SSTR antagonist, TKi, Everolimus	0	2	Bone, liver, LNM	None
**30**	M	76	Small intestine	G1	3	Surgery, liver TACE, SST analogue, radiotherapy, beta-PRRT with SSTR agonist	1	0	Bone, liver	Lost during follow-up
**31**	M	53	Pancreas	G3	60	Surgery, liver TACE, SST analogue, chemotherapy, radiotherapy, beta-PRRT with	1	0	Bone, liver, LNM	Anemia G2

						SSTR agonist, beta-PRRT with SSTR antagonist, TKi, Everolimus				
**32**	F	48	Pancreas	G3	30	Surgery, SST analogue, chemotherapy, beta-PRRT with SSTR antagonist	0	1	Bone, liver	Thrombocytopenia G3
**33**	M	65	Small intestine	G2	12	Surgery, SST analogue, beta-PRRT with SSTR agonist	0	1	Bone, liver, LNM	None
**34**	F	39	Ovary	G3	35	Surgery, SST analogue, chemotherapy, radiotherapy, Nivolumab	1	0	Bone, liver, LNM	Leukocytopenia G4, thrombocytopenia G3
**35**	M	66	Small intestine	G3	40	Liver TACE, SST analogue, chemotherapy, beta-PRRT with SSTR agonist, TKi, Everolimus	1	0	Bone, liver	Lost during follow-up

*Age at first alpha-DOTA-LM3 PRRT (either monotherapy or TANDEM).

Legend: M: male; F: female; PRRT: Peptide Receptor Radionuclide Therapy; SST: somatostatin; SSTR: somatostatin receptor; TKi: tyrosine-kinase inhibitors; ANC: absolute neutrophilic count; AML: acute myeloid leukemia; MIBG: metaiodobenzylguanidine; LNM: lymph node metastases; n.r.: not reported.
